# Clinical importance of the range of detectable variants between the Oncomine Dx target test and a conventional single-gene test for EGFR mutation

**DOI:** 10.1038/s41598-023-40271-w

**Published:** 2023-08-23

**Authors:** Tadashi Sakaguchi, Akemi Iketani, Seiya Esumi, Maki Esumi, Yuta Suzuki, Kentaro Ito, Kentaro Fujiwara, Yoichi Nishii, Koji Katsuta, Hiroki Yasui, Osamu Taguchi, Osamu Hataji

**Affiliations:** 1 Department of Respiratory Medicine, Matsusaka Municipal Hospital, 1550, Tonomachi, Matsusaka, Mie 515-0073 Japan; 2 Pathology Department, Matsusaka Municipal Hospital, 1550, Tonomachi, Matsusaka, Mie 515-0073 Japan

**Keywords:** Cancer, Molecular medicine, Oncology

## Abstract

Although we have experienced some cases with discordant results between the Oncomine Dx target test (ODxTT) and conventional single gene tests for detecting *EGFR* alterations, the clinical efficacy of EGFR-TKIs in these discordant cases remains little known. We retrospectively reviewed consecutive patients with non-small-cell lung cancer whose FFPE samples were simultaneously submitted for the ODxTT, and a PNA-LNA PCR clamp test. We evaluated the clinical efficacy of EGFR-TKIs in patients with discordant results between the two tests, focusing on the common *EGFR* mutations. Among 444 successful results, 10 patients had discordant results for common *EGFR* mutations (9 Ex 19 deletion and 1 Ex 21 L858R mutation), and all of these were detected only by the PNA-LNA PCR clamp test. Among six discordant cases treated with EGFR-TKI, the mutations detected in 3 patients were not included in the list of detectable variants that are reportable by the ODxTT, while the mutations detected in the other 3 patients were included in the list. For all three discordant cases harboring the mutations not reportable by the ODxTT, good clinical responses were demonstrated. However, among the other three discordant cases harboring the mutations reportable by the ODxTT, only one patient had a clinical response with short duration. Among the discordant cases for common *EGFR* mutations between the ODxTT and the conventional single gene test, there are a certain number of suitable patients responsive to EGFR-TKIs, especially when the cause of the discordant results comes from the difference in the range of detectable variants that are reportable between the tests.

## Introduction

Various molecular-targeted drugs for patients with non-small-cell lung cancer (NSCLC) harboring driver oncogene alterations have improved patient prognoses better than conventional cytotoxic chemotherapy^1–5^. Clinical guidelines therefore recommend that patients with advanced NSCLC harboring driver oncogene alterations receive targeted therapies for each alteration as the first-line treatment^6–8^. Among the driver oncogene alterations, epidermal growth factor receptor (*EGFR*) alterations are most commonly detected, occurring in 45% of patients with advanced non-squamous NSCLC in Japan^9^.

Conventional single-gene tests for *EGFR* mutations, such as the cobas *EGFR* assay, the therascreen *EGFR* assay as an in vitro diagnostic (IVD) test, and the peptide nucleic acid-locked nucleic acid polymerase chain reaction (PNA-LNA PCR) clamp assay as a laboratory-developed test (LDT), have been widely used as single companion diagnostic tests for EGFR-tyrosine kinase inhibitors (TKIs)^10–12^. However, as more target genes have been identified and targeted therapies have been approved in clinical settings, comprehensive biomarker testing has become necessary to make appropriate treatment decisions.

Next-generation sequencing (NGS) can detect multiple gene variants simultaneously, enabling comprehensive genetic testing. The Oncomine Dx target test (ODxTT) (Ion Torrent PGM Dx Sequencer; Thermo Fisher Scientific) is one of the NGS panels, and was approved by the US Food and Drug Administration in June 2017^13^. Since February 2019, this test has also been approved in Japan as a companion diagnostic for targeted therapies on 4 driver alterations: *EGFR* mutations, *ALK* fusion genes, *ROS1* fusion genes, and *BRAF* mutation (p.V600E). In addition, *RET* fusion genes have been added to the ODxTT as a companion diagnostic in Japan since September 2021.

Although we have previously reported some cases with discordant results between the ODxTT and the PNA-LNA PCR clamp assay for the detection of EGFR mutations^14^, the clinical efficacy of EGFR-TKIs in these discordant cases remains little known. Therefore, in this study, we retrospectively evaluated the clinical efficacy of EGFR-TKIs in patients with discordant results between the two tests, focusing on the common EGFR mutations (Ex 19 deletion and Ex 21 L858R), taking into account the identified variants of the mutations.

## Results

### Success and detection rates of *EGFR* mutations for ODxTT and PNA-LNA PCR clamp test

**Figure 1:**
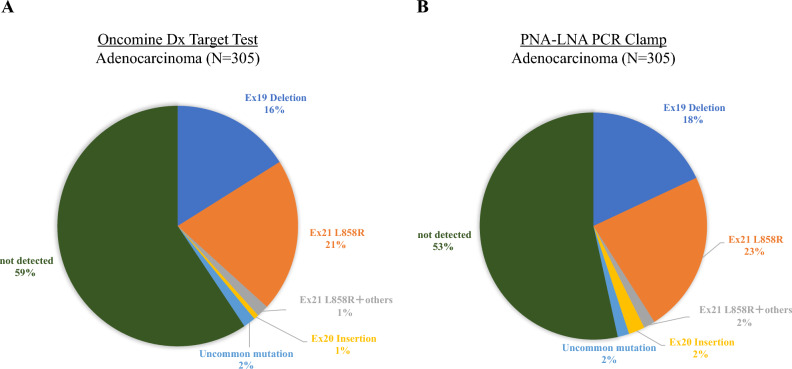
The detection rates of *EGFR* mutations for adenocarcinoma. (**A**) Detection rate of Oncomine Dx target test. (**B**) Detection rate of PNA-LNA PCR clamp test.

A total of 470 samples were identified for comparison analysis. The sample characteristics for the analysis are shown in Table 1. The success rates for the ODxTT and PNA-LNA PCR Clamp test are shown in Table 2. Although the success rate of the ODxTT was 94% (95% CI 92.0–96.4), the success rate of the PNA-LNA PCR Clamp test was 100% (95% CI 99.2–100). The detection rates for *EGFR* mutations of each test for adenocarcinoma, are shown in Fig. 1. The detection rate of the ODxTT was 41% (95% CI 35.1–46.4), and the PNA-LNA PCR Clamp test was 47% (95% CI 40.9–52.3) (P < 0.01). Among the 26 samples unsuccessfully analyzed by ODxTT, six Ex 21 L858R mutations were detected by the PNA-LNA PCR Clamp test.

**Table 1:** Sample characteristics.

	Total samples	
	N = 470	(%)
Sampling method		
EBB/TBB	234	50
Surgical resection	110	23
CTNB	103	22
Pleural biopsy	11	2
Others	12	3
Histology		
ADC	305	65
Sq	121	26
Non-Sq non-ADC	23	5
NSCC NOS	21	4

*EBB* endobronchial biopsy, *TBB* transbronchial biopsy, *CTNB* computed tomography-guided needle aspiration, *ADC* adenocarcinoma, *Sq* squamous cell carcinoma, *NSCC NOS* non-small cell carcinoma, not otherwise specified.

**Table 2:** Analysis success rates of ODxTT and PNA-LNA PCR clamp test.

	Total samples N = 470			
	ODxTT	(%)	PNA-LNA PCR clamp	(%)
Success of analysis	444	94	470	100
Not passing the nucleic acid concentration threshold	10	2	0	0
Invalid results for all *EGFR* mutations	8	2	0	0
Invalid results for subset of *EGFR* mutations	8	2	0	0

*EGFR* epidermal growth factor receptor, *ODxTT* Oncomine Dx target test.

Among the 444 samples successfully analyzed by both tests, 10 discordant results for common EGFR mutations were reported, whose clinical characteristics and courses are shown in Table 3. The ODxTT failed to detect 9 Ex 19 deletion and 1 Ex 21 L858R mutation that could be detected with the PNA-LNA PCR Clamp test. For the discordant cases, the additionally performed direct sequence methods identified the 5 specific variants recorded in the Catalogue of Somatic Mutations in Cancer (COSMIC) database [COSMIC ID: 6224, 6225, 12382, 12678, 133196] in 6 cases, while the variants of the other cases were not recorded in the COSMIC database. 4 specific variants with an assigned COSMIC ID [6224, 6225, 12382, 12678] are included in the list of detectable variants that can be reportable by ODxTT, however the other variants are not included in the list.

**Table 3:** Clinical characteristics and course of discordant cases.

No	Age	Sex	Histology	Result of EGFR mutation			TPS (%)	Stage (UICC-8)	EGFR-TKI	Clinical response
				ODxTT	PNA-LNA PCR clamp	COSMIC ID				
1	47	M	ADC	Negative	Ex 19 del	6225	1–5	IVB	Osimertinib	PD
2	70	M	LCC	Negative	Ex 19 del	12,678	1	Postoperative recurrence	Osimertinib	PR
3	68	M	ADC	Negative	Ex 19 del	Not registered	< 1	IA3	Not administered	NA
4	68	F	ADC	Negative	Ex 19 del	Not registered	10–20	Postoperative recurrence	Osimertinib	PR
5	77	M	ADC	Negative	Ex 21 L858R	6224	10–20	IVB	ERL + BEV	PD
6	69	M	ADC	Negative	Ex 19 del	133,196	1–5	IVB	Osimertinib	PR
7	70	F	ADC	Negative	Ex 19 del	Not registered	100	IVB	Osimertinib	PR
8	91	M	NSCC NOS	Negative	Ex 19 del	12,382	90	IVB	Not administered	NA
9	50	F	ADC	Negative	Ex 19 del	Not registered	< 1	IIB	Not administered	NA
10	78	M	Sq	Negative	Ex 19 del	12,678	70–80	IB	Not administered	NA

*ADC* adenocarcinoma, *LCC* large cell carcinoma, *NSCC NOS* non-small cell carcinoma, not otherwise specified, *Sq* squamous cell carcinoma, *EGFR* epidermal growth factor receptor, *ODxTT* Oncomine Dx target test, *COSMIC* catalogue of somatic mutations in cancer, *TPS* tumor proportion score, *UICC* Union for International Cancer Control, *TKI* tyrosine kinase inhibitor, *ERL* erlotinib, *BEV* bevacizumab, *PD* progression disease, *PR* partial response, *NA* not assessed.

### Clinical efficacy of EGFR-TKI therapy for the discordant cases

**Figure 2:**
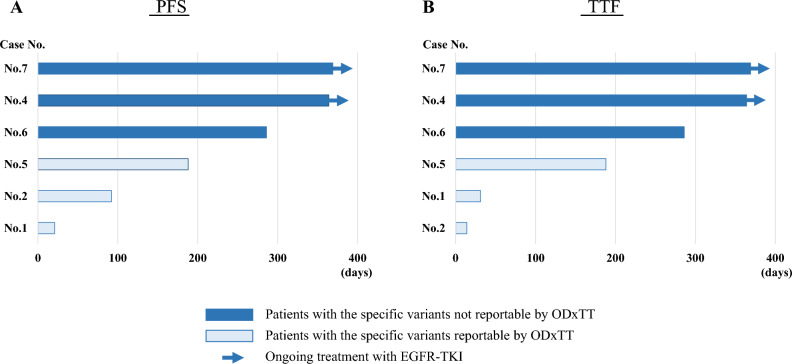
The swimmer plots of PFS (**A**) and TTF (**B**) of EGFR-TKI for discordant cases categorized into two variant groups related to the detectable range of both tests. *PFS* progression free survival, *TTF* time to treatment failure, *EGFR-TKI* epidermal growth factor receptor-tyrosine kinase inhibitor, *ODxTT* Oncomine Dx target test.

Six of the ten discordant cases of common EGFR mutations were treated with EGFR-TKIs (5 with osimertinib, and 1 with erlotinib plus bevacizumab), all of whom received the treatments as first line therapy. Among the three treated patients harboring the common EGFR mutations not reportable by ODxTT, all of them had good clinical responses. However, among the other three treated patients harboring the common EGFR mutations reportable by ODxTT, only one patient had clinical response with short duration. PFS and TTF of the patients harboring the common EGFR mutations not reportable by ODxTT were longer than those of the patients harboring the common EGFR mutations reportable by ODxTT (Fig. 2). Of the six cases treated with EGFR TKIs, only one case (No. 5) had a concurrent KRAS mutation, whereas the other cases did not have a significant concurrent alteration detectable by ODxTT, such as a PIK3CA mutation. Each allele frequency (AF) of the specific variant reportable by ODxTT in the three discordant cases was zero.

## Discussion

In recent years, multi-gene tests able to detect multiple driver alterations simultaneously, such as NGS and PCR panel tests, have become available in clinical practice. Although we have experienced some cases with discordant results between the multi-gene tests and conventional single-gene tests in such situations^14,16^, the clinical utility of targeted therapies for these discordant cases remains little known. To our knowledge, this is the first report to assess the clinical efficacy of EGFR-TKIs in patients with discordant results for common EGFR mutations between the ODxTT and a single gene test, focused on the range of detectable variants in each test.

In this study, all common EGFR mutations in the discordant results were only detected with the PNA-LNA PCR clamp test, and we additionally ordered the laboratories to perform the direct sequence method using reaction products after the PNA-LNA PCR clamp assay to identify the COSMIC ID of the specific variants. As for the cause of the discordant results, two main reasons should be considered. As shown in our previous report, one is the difference in limit of detections (LODs) between the ODxTT and the PNA-LNA PCR Clamp test, and the other is the difference in the range of detectable variants for each test. The estimated LODs of the ODxTT were reported to be 6% AF for *EGFR* Ex 19 deletion, and 8% AF for Ex 21 L858R^17^. A clinical bridging study to establish the assurance of the ODxTT compared with the therascreen *EGFR* assay, whose LODs were 1–2% for *EGFR* common mutations, was performed by Thermo Fisher Scientific and showed good concordance^17^. Meanwhile, the LODs for the PNA-LNA PCR Clamp test are estimated to be 0.1–1%^11^, therefore it is expected that the PNA-LNA PCR clamp test can detect lower AFs for *EGFR* mutations. If the discordant results come from the difference in LOD between the tests, the tumors harboring the mutation would not be predominant in the whole tumor when the assumed tumor content was not overestimated, therefore poor EGFR-TKI efficacy is expected in these cases. If the discordant results come from the difference in the range of detectable variants of each test the tumors harboring the mutation may be predominant in the whole tumor, therefore good EGFR-TKI efficacy is expected in these cases. Among the three patients in this study treated with EGFR-TKIs harboring common EGFR mutations reportable by ODxTT, this indicating the discordant results were due to the difference of LOD between the both tests, only one patient had a clinical response with short duration. Meanwhile, among the three patients treated with EGFR-TKI, harboring Ex 19 deletion variants not reportable by ODxTT, this indicating the discordant results were due to the difference in the range of detectable variants between the tests, all of them had good clinical responses with long duration.

EGFR Ex 19 deletions consist of distinct molecular variants and represent a heterogeneous disease entity, of which the most common Ex 19 deletion variant is ΔE746_A750 deletion, detected in 65–72% of patients with EGFR Ex 19 deletion variants^18,19^. Although the efficacy of EGFR-TKIs for uncommon (non-ΔE746_A750 deletion) Ex 19 deletion variants is controversial, some previous reports have demonstrated good efficacy of EGFR-TKIs among patients harboring uncommon Ex19 deletion variants^20,21^. Our results suggested that EGFR-TKIs would be a suitable treatment option for patients harboring uncommon Ex19 deletion variants, and missing suitable patients responsive to EGFR-TKIs due to the difference in range of detectable variants for each test would be an important issue to be avoided. Therefore, the ability of a test to detect and report an extensive range of mutation variants would be a desired factor in optimal genetic testing.

There were several limitations to this study. First, this study was a relatively small retrospective study, which resulted in an analysis with a small number of cases. A larger sample size would be needed to evaluate the frequency of discordant cases, and the clinical meaning of detecting the discordant cases considering the clinical benefit of targeted therapies. Second, tumor content was evaluated subjectively by skilled cytopathologists in our institution, but not objectively by artificial intelligence or other means. Previous reports suggest that pathologists often overestimate the contents of tumor cells, and the use of AI-based analysis increases the accuracy^22^. Therefore, it is difficult to precisely evaluate the relation between clinical benefit and tumor content in this study, although the tumor content plays a large role in the interpretation of the AF. Third, this study could not adequately evaluate other predictors potentially associated with EGFR-TKI efficacy, such as concurrent genetic alterations including TP53 mutation and PTEN alteration^23^, because the ODxTT could not evaluate these genes. Further studies, including evaluation for such predictors with larger genetic panel tests, would be needed. Finally, the range of detectable and reportable mutation variants was different for each single-gene and multi-gene test; therefore, the results of this study are not applicable to other single-gene and multi-gene tests. In conclusion, among the discordant cases between the ODxTT and conventional single-gene tests, a certain number of suitable patients are responsive to EGFR-TKIs, especially when the discordant results come from the difference in the range of detectable or reportable variants in each test.

## Materials and methods

### Patient selection

This retrospective study was conducted at Matsusaka Municipal Hospital, Japan. We reviewed the electronic data from consecutive NSCLC patients whose formalin-fixed and paraffin-embedded (FFPE) samples were simultaneously submitted for an ODxTT, and a PNA-LNA PCR clamp test, using the same specimen, from August 2019 to March 2022. Samples collected in other hospitals, and archived samples were excluded. Clinical data assessments included; patient characteristics, sampling methods, staging, histology, pathological findings, the results of genetic tests, and the clinical course with EGFR-TKI treatment. This study was performed in accordance with the Declaration of Helsinki. This study was approved by the institutional review board of Matsusaka Municipal Hospital (IRB number J-76-200410-5-2). Informed consent was obtained by opt-out method.

### Sample processing and genetic tests

The FFPE sample processing methods of our institution were shown in detail in our previous reports^14,15^. The amount of tumor cells, and the tumor content of the sample stained with hematoxylin and eosin, were evaluated by skilled cytopathologists. In some samples obtained since 2020, macro-dissection was performed as needed in our institution. If the tumor content was < 20% after marking and macro-dissection, or the amount of tumor cells was insufficient, the sample was not submitted for the ODxTT. For the ODxTT, 10 to 20 slide-mounted 5 to 10-µm sections of small biopsy samples, and 5 to 10 slide-mounted 5 to 10-µm sections of surgical resection samples, depending on each sample volume, were submitted to LSI Medience Laboratories (Tokyo, Japan). For the PNA-LNA PCR clamp test, 5 slides of 5-µm sections from small biopsy samples and surgical resection samples were submitted to the laboratories. LSI Medience Laboratories performed the ODxTT based on Thermo Fisher’s Ion AmpliSeq technology, and the PNA-LNA PCR Clamp tests were performed using the PNA-LNA PCR clamp assay^11,13^. When a discordant result whose EGFR mutation was only detected by PNA-LNA PCR clamp assay was returned, we additionally ordered the laboratories to perform the direct sequence method using reaction products after the PNA-LNA PCR clamp assay to identify the specific variants of EGFR mutations.

### Specific *EGFR* mutations and mutation variants detectable by each test

The specific *EGFR* mutations detectable by the ODxTT and PNA-LNA PCR Clamp test were mentioned in our previous report^14^. In addition, the range of detectable EGFR mutation variants for the ODxTT is publicly available, and shown in Supplemental Table 1. Although the range of the PNA-LNA PCR Clamp test was not published due to LDT, the test was designed to be able to detect an extensive range of variants.

### Outcomes

We evaluated the success rate and the detection rate of *EGFR* mutations for the ODxTT compared with those of the PNA-LNA PCR clamp test in the same manner as shown in our previous report^14^. In this study, focused on the NSCLC patients with discordant common EGFR mutation results between the ODxTT and the PNA-LNA PCR clamp test, we evaluated their clinical response, progression-free survival (PFS), and the time to treatment failure (TTF) of EGFR-TKIs.

The analysis results were regarded as successful if all results for the following *EGFR* mutations reported for each time period were completely available; exon 18 (p.G719A/C/S), exon 19 (deletion), exon 20 (p.S768I, p.T790M, insertion), and exon 21 (p.L858R and p.L861Q). These being the mutations considered required for detection due to clinical implications by the Japanese Lung Cancer Society. Meanwhile, the analysis results were regarded as unsuccessful if the sample did not pass the nucleic acid concentration threshold, or if one or more of the *EGFR* mutation results mentioned above were invalid due to a failure to meet the DNA sample quality control (QC) metrics, or no call.

### Statistical analysis

Statistical analyses were performed using Pearson’s Chi-squared test for comparison of analysis success rates and detection rates, and 95% Clopper–Pearson confidence interval (CI) were calculated. Statistical significance was indicated with a P-value less than 0.05. Statistical analyses were performed using SPSS software, version 26.0 (SPSS Inc., Chicago, USA).

### Supplementary Information

Supplementary Table 1.

## Data Availability

The data that support the findings of this study are available from the corresponding author upon reasonable request.

## References

[1] Maemondo M (2010). Gefitinib or chemotherapy for non-small-cell lung cancer with mutated EGFR. N. Engl. J. Med..

[2] Zhou C (2011). Erlotinib versus chemotherapy as first-line treatment for patients with advanced EGFR mutation-positive non-small-cell lung cancer (OPTIMAL, CTONG-0802): A multicentre, open-label, randomised, phase 3 study. Lancet Oncol..

[3] Yang JC (2015). Afatinib versus cisplatin-based chemotherapy for EGFR mutation-positive lung adenocarcinoma (LUX-Lung 3 and LUX-Lung 6): Analysis of overall survival data from two randomised, phase 3 trials. Lancet Oncol..

[4] Solomon BJ (2014). First-line crizotinib versus chemotherapy in ALK-positive lung cancer. N. Engl. J. Med..

[5] Soria J-C (2017). First-line ceritinib versus platinum-based chemotherapy in advanced ALK-rearranged non-small-cell lung cancer (ASCEND-4): A randomised, open-label, phase 3 study. The Lancet.

[6] Ettinger DS (2022). NCCN Clinical Practice Guidelines in Oncology: Non-small cell lung cancer. Version 3. J. Natl. Compr. Cancer Netw..

[7] Planchard D (2018). Metastatic non-small cell lung cancer: ESMO clinical practice guidelines for diagnosis, treatment and follow-up. Ann. Oncol..

[8] Akamatsu H (2019). The Japanese Lung Cancer Society guideline for non-small cell lung cancer, stage IV. Int. J. Clin. Oncol..

[9] Midha A, Dearden S, McCormack R (2015). EGFR mutation incidence in non-small-cell lung cancer of adenocarcinoma histology: A systematic review and global map by ethnicity (mutMapII). Am. J. Cancer Res..

[10] *Therascreen® EGFR RGQ PCR Kit Handbook Version 1*. https://www.qiagen.com/ch/resources/download.aspx?id=db794cae-999b-4362-aba3-455ebfd807a5&lang=en.

[11] Nagai Y (2005). Genetic heterogeneity of the epidermal growth factor receptor in non-small cell lung cancer cell lines revealed by a rapid and sensitive detection system, the peptide nucleic acid-locked nucleic acid PCR clamp. Cancer Res..

[12] Kimura H (2014). Analytical performance of the cobas EGFR mutation assay for Japanese non-small-cell lung cancer. Lung Cancer.

[13] Food and Drug Administration. *Oncomine*^*TM*^* Dx Target Test Part I: Sample Preparation and Quantification User Guide. Revision C.0 2017*. https://www.accessdata.fda.gov/cdrh_docs/pdf16/P160045C.pdf (Accessed 14 June 2020).

[14] Sakaguchi T (2021). Comparison of the analytical performance between the Oncomine Dx target test and a conventional single gene test for epidermal growth factor receptor mutation in non-small cell lung cancer. Thorac. Cancer.

[15] Sakaguchi T (2021). A method to improve genetic analysis of lung cancer samples. Respirology.

[16] Murakami S (2022). Comparison of next-generation sequencing and cobas EGFR mutation test v2 in detecting EGFR mutations. Thorac. Cancer.

[17] Food and Drug Administration. *Summary of Safety and Effectiveness Data 2017*. https://www.accessdata.fda.gov/cdrh_docs/pdf16/P160045B.pdf (Accessed 26 October 2022).

[18] Zhao C (2020). The impact of EGFR exon 19 deletion subtypes on clinical outcomes in non-small cell lung cancer. Transl. Lung Cancer Res..

[19] Tokudome N (2020). Differential significance of molecular subtypes which were classified into EGFR exon 19 deletion on the first line afatinib monotherapy. BMC Cancer.

[20] Peng X (2020). Clinical impact of uncommon epidermal growth factor receptor exon 19 insertion–deletion variants on epidermal growth factor receptor–tyrosine kinase inhibitor efficacy in non-small-cell lung cancer. Eur. J. Cancer.

[21] Rossi S (2019). Impact of exon 19 deletion subtypes in EGFR-mutant metastatic non-small-cell lung cancer treated with first-line tyrosine kinase inhibitors. Clin. Lung Cancer.

[22] Sakamoto T (2020). A narrative review of digital pathology and artificial intelligence: Focusing on lung cancer. Transl. Lung Cancer Res..

[23] Guo Y (2020). Concurrent genetic alterations and other biomarkers predict treatment efficacy of EGFR-TKIs in EGFR-mutant non-small cell lung cancer: A review. Front. Oncol..

