# Supplementary material for: Clinical importance of the range of detectable variants between the Oncomine Dx target test and a conventional single-gene test for EGFR mutation
Source: Sci Rep. 2023 Aug 23;13:13759. doi: 10.1038/s41598-023-40271-w (PMC10447417; doi:10.1038/s41598-023-40271-w)
Supplement: Supplementary file 1 — Supplementary Table 1. [file 41598_2023_40271_MOESM1_ESM.pdf]

Supplemental Table 1. The list of EGFR variants detectable by the ODxTT as a companion diagnostic.

Exon No.	COSMIC ID	Amino Acid Change	Nucleotide Change
18	COSM12988	p.E709K	c.2125G>A
18	COSM13427	p.E709A	c.2126A>C
18	COSM13009	p.E709G	c.2126A>G
18	COSM12371	p.E709V	c.2126A>T
18	COSM6252	p.G719S	c.2155G>A
18	COSM6253	p.G719C	c.2155G>T
18	COSM18425	p.G719D	c.2156G>A
18	COSM6239	p.G719A	c.2156G>C
19	COSM26038	p.K745_E749del	c.2233_2247delAAGGAATTAAGAGAA
19	COSM1190791	p.K745_A750delinsT	c.2234_2248delAGGAATTAAGAGAAG
19	COSM28517	p.E746_E749del	c.2235_2246delGGAATTAAGAGA
19	COSM6223	p.E746_A750del	c.2235_2249delGGAATTAAGAGAAGC
19	COSM13551	p.E746_T751delinsI	c.2235_2252delGGAATTAAGAGAAGCAACinsAAT
19	COSM6225	p.E746_A750del	c.2236_2250delGAATTAAGAGAAGCA
19	COSM12728	p.E746_T751del	c.2236_2253delGAATTAAGAGAAGCAACA
19	COSM12678	p.E746_T751delinsA	c.2237_2251delAATTAAGAGAAGCAA
19	COSM12416	p.E746_T751delinsVA	c.2237_2253delAATTAAGAGAAGCAACAinsTTGCT
19	COSM12384	p.E746_S752delinsV	c.2237_2255delAATTAAGAGAAGCAACATCinsT
19	COSM12422	p.L747_A750delinsP	c.2238_2248delATTAAGAGAAGinsGC
19	COSM12419	p.L747_T751delinsQ	c.2238_2252delATTAAGAGAAGCAACinsGCA
19	COSM6220	p.E746_S752delinsD	c.2238_2255delATTAAGAGAAGCAACATC
19	COSM6218	p.E746_R748del	c.2239_2247delTTAAGAGAA
19	COSM12382	p.L747_A750delinsP	c.2239_2248delTTAAGAGAAGinsC
19	COSM12383	p.L747_T751delinsP	c.2239_2251delTTAAGAGAAGCAAinsC
19	COSM6255	p.L747_S752del	c.2239_2256delTTAAGAGAAGCAACATCT
19	COSM12387	p.L747_P753delinsQ	c.2239_2258delTTAAGAGAAGCAACATCTCCinsCA
19	COSM6210	p.L747_T751delinsS	c.2240_2251delTAAGAGAAGCAA
19	COSM12369	L747_T751del	2240_2254del15
19	COSM12370	p.L747_P753delinsS	c.2240_2257delTAAGAGAAGCAACATCTC
20	COSM6241	p.S768I	c.2303G>T
20	COSM6240	p.T790M	c.2369C>T
21	COSM6224	p.L858R	c.2573T>G
21	COSM6213	p.L861Q	c.2582T>A
21	COSM12374	p.L861R	c.2582T>G

Abbreviations: COSMIC, Catalogue of Somatic Mutations in Cancer.
